# Genome-Wide Organization and Expression Profiling of the SBP-Box Gene Family in Chinese Jujube (*Ziziphus jujuba* Mill.)

**DOI:** 10.3390/ijms18081734

**Published:** 2017-08-15

**Authors:** Shuang Song, Heying Zhou, Songbai Sheng, Ming Cao, Yingyue Li, Xiaoming Pang

**Affiliations:** 1 Beijing Advanced Innovation Center for Tree Breeding by Molecular Design, National Engineering Laboratory for Tree Breeding, Key Laboratory of Genetics and Breeding in Forest Trees and Ornamental Plants, Ministry of Education, College of Biological Sciences and Biotechnology, Beijing Forestry University, Beijing 100083, China; shuangsong@bjfu.edu.cn (S.S.); zhydyx2012@126.com (H.Z.); shengsongbai312@139.com (S.S.); yingyueli@bjfu.edu.cn (Y.L.); 2 National Foundation for Improved Cultivar of Chinese Jujube, Cangzhou 061000, China; i2008caoming@126.com

**Keywords:** transcription factors, SBP gene family, *Ziziphus jujuba* Mill., Dongzao, expression profile

## Abstract

Transcription factors play vital roles in the developmental processes of plants. The SQUAMOSA promoter binding protein (SBP) genes encode a family of plant-specific transcription factors and plays many crucial roles in plant development. In this study, 16 SBP-box gene family members were identified in *Ziziphus jujuba* Mill. Dongzao (Dongzao), which were distributed over 8 chromosomes. They were classified into seven groups according to their phylogenetic relationships with other SBP-box gene families. Within each group, genes shared similar exon-intron structures and motif locations. The number of exons varied among the groups. We identified 12 homologous gene pairs between Dongzao and *Arabidopsis*. Expression profiling revealed that *ZjSBP02* and *ZjSBP14* expressed highly in mature fruits, *ZjSBP01* expressed higher in mature leaves than other tissues and the expression level of *ZjSBP12* was much higher in the flowers. The transcriptome analysis indicated that ZjSBPs had different expression patterns in various tissues. This study represents the first systematic analysis of the SBP-box gene family in *Z. jujuba*. The data presented here provides a foundation for understanding the crucial roles of *ZjSBP* genes in plant growth and development.

## 1. Introduction

Transcription factors are proteins that bind DNA in a sequence-specific way and activate or repress the transcription of target genes to regulate the specific expression in different tissues and different environments. In plants, the transcription factors play important roles during the development process. The SBP-box genes encode a family of transcription factors that are specific in plants, which have a highly conserved domain with residues of about 76 amino acids. This conserved domain contains DNA-binding and nuclear localization with two zinc-binding sites [1,2,3]. SBP-box genes (*AmSBP1* and *AmSBP2*) were first identified in *Antirrhinum majus* [4], based on their ability to interact with the promoter sequence region of the floral meristem identity gene, SQUAMOSA [1] (Klein et al., 1996). The SBP-box family has been identified in many plants, such as *Arabidopsis* [5], rice [6] and apple [7] etc.

Alternative splicing is a regulated process during gene expression that can code multiple proteins from one gene. There were 16 members in the *Arabidopsis* genome, of which nine were alternative splicing genes, such as At1g02065, At1g27360 and At1g27370 [2,5]. *SPL3* (SQUAMOSA promoter binding protein-like 3) was demonstrated to play a significant role in floral transition [8] and most other members have also been explored [5,6] with functions involving sporogenesis [9], bud and leaf development [10,11], flowering [12], vegetative and reproductive phase transitions [13] and plant hormone signaling [14]. In rice, 19 members have been identified. More than half of the *OsSPL* genes were mainly expressed in young panicles [6]. *OsSPL14* [15] and *OsSPL16* [16] were demonstrated to play important roles in determining grain quality and yield in rice. There were 27 SBP-box gene family members in the apple genome and 15 of them were suggested to be putative targets of MdmiR156 [7]. In the tomato, there were 15 SBP-box gene family members. All the SlySBPs could be detected in these apical regions and be related to the response of specific stress. Most miR156-targeted SlySBPs expressed a high level in young inflorescences and during fruit development and ripening, suggesting that it plays an important role during tomato reproductive growth [17]. Manning et al. 2006 [18] used positional cloning and virus-induced gene silencing to confirm the SBP-box gene-*CNR* is strategic for normal ripening [18,19,20]. So far, there were 18 SBP-box gene family members in the grape and 12 of them were complementary to miRNA156/157. Some *VvSBP* genes could prospectively participate in the defense against biotic and abiotic stresses [21].

Chinese jujube (*Ziziphus jujuba* Mill.) (2n = 2x = 24), which belongs to the Rhamnaceae family, is estimated to have been cultivated for more than 7000 years [22]. Its original cultivation center is in the middle and lower reaches of the Yellow River [22]. Chinese jujube is cultivated in all the provinces of China except Heilongjiang and Tibet and the total planted area was about 2 million hectares [23,24]. So far, Chinese jujube has been introduced to more than 50 countries, such as Korea, Japan, Russia, India, Thailand, Europe, the United States and Australia [25]. Jujube fruit is abundant in biologically active components like vitamin C, phenolics, avonoids, triterpenic acids and polysaccharides, and is considered to have important medicinal uses [26]. Its fruit can be consumed as fresh fruit, dried fruit or processed into various jujube products [27].

Although the SBP-box gene family has been identified in many plants, nothing is known about the SBP-box gene family in the Chinese jujube. In this study, we identified 16 characterized putative SBP-box genes in the jujube genome and the expression pattern of *ZjSBP* genes in different tissues or organs were also surveyed. The outcomes of this study could facilitate further dissection of the SBP-box gene family in jujube.

## 2. Results

### 2.1. Identification of the SBP-Box Gene Family in Dongzao

**Figure 1:**
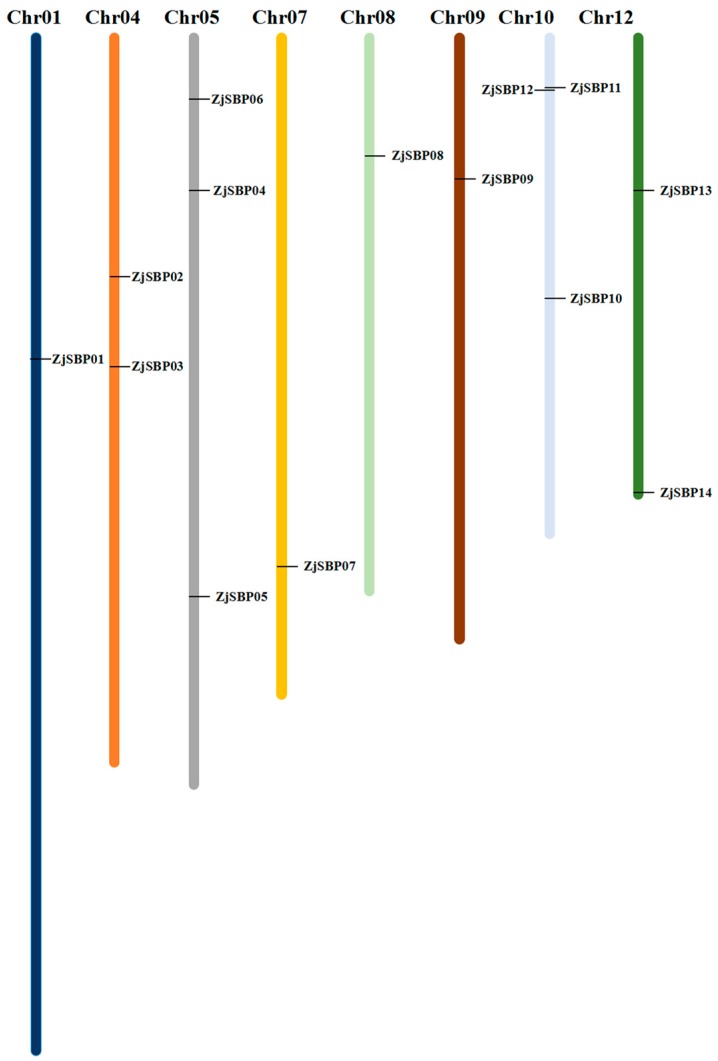
Distribution of *ZjSBP* genes over the jujube chromosomes.

We used the *AtSBP* genes to identify the gene family members on the NCBI. After blast in jujuba genome, we reorganized and merged the highly matched sequences and got 37 sequences that satisfied the requirement. InterProScan was used to scan for the SBP domain and to find the final members. Seventeen of them were excluded for having the same sequences and one of them was removed due to an incomplete SBP domain. There were 28 putative SBP genes in jujube as shown in Table 1, including seven alternative splicing genes (LOC107418038, LOC107423307, LOC107426205, LOC107428311, LOC107428287, LOC107432336 and LOC107408850). The sequences of each of the alternative splicing genes were similar, so we chose the longest transcript for the following analysis. The deduced length of the SBP proteins ranged from 154 (ZjSBP01) to 1059 (ZjSBP02) amino acids, while the pI values ranged from 5.20 (ZjSBP08) to 9.56 (ZjSBP15), which suggests that different SBP proteins might operate in different microenvironments. The putative SBP genes mapped to the chromosomes shown in Figure 1, provide a viewable insight into SBP-box gene distribution. According to the most recently assembled jujube genome resources [28], 16 ZjSBPs were unevenly distributed over 8 chromosomes with one on each of Chr01, Chr07, Chr08 and Chr09, two on each of Chr04 and Chr12, and three on each of Chr05 and Chr10. Only ZjSBP15 and ZjSBP16 were not assigned to any chromosomes.

### 2.2. Sequence Alignments and Phylogenetic Analyses

**Figure 2:**
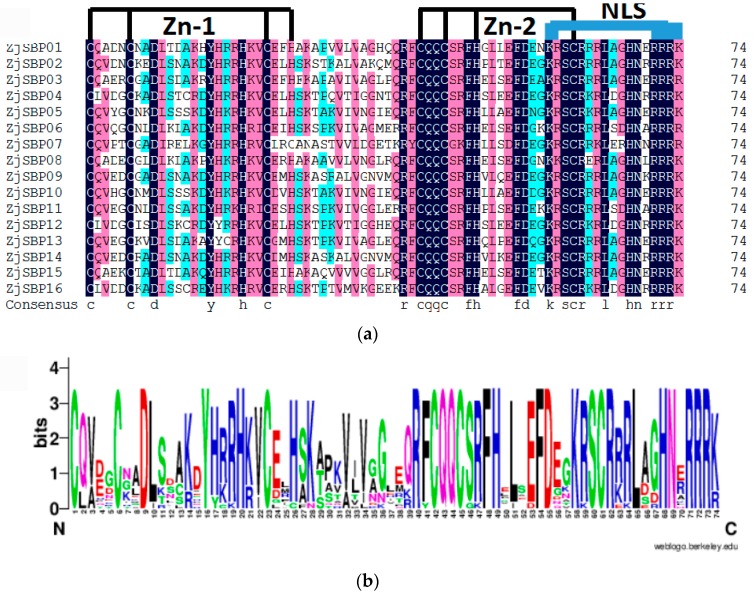
SBP domain alignment of the ZjSBPs. (**a**) Multiple alignments of the SBP domains of the ZjSBPs by *DNAMAN*; the two conserved zinc finger structures (Zn-1, Zn-2) and *NLS* are indicated; (**b**) Sequence logos of the SBP domain by the online Gene Structure Display Server; the overall height of each stack represents the degree of conservation at this position, while the height of the letters within each stack indicates the relative frequency of the corresponding amino acids.

Multiple sequence alignment of full length protein sequences was completed by the DNAMAN (version 6.0, Lynnon Corp., Quebec, QC, Canada, http://www.lynnon.com/index.html) for defining the structure of each gene. There was one conserved SBP domain in all members (Figure 2a). These SBP domains were highly conserved at some positions, such as CQQC sequences, SCR sequences and RRR sequences (Figure 2b). Interestingly, all members have two zinc finger-like structure-Zn-1, Zn-2 and a highly conserved bipartite nuclear localization signal (NLS). The nuclear localization signal was partly overlapped with the second zinc finger-like structure [2]. All the ZjSBPs possessed the same zinc finger-like structure CysCysHisCys, while CysCysCysHis was another zinc finger-like structure for all members except ZjSBP07 with a zinc finger-like structure CysCysCysCys.

**Figure 3:**
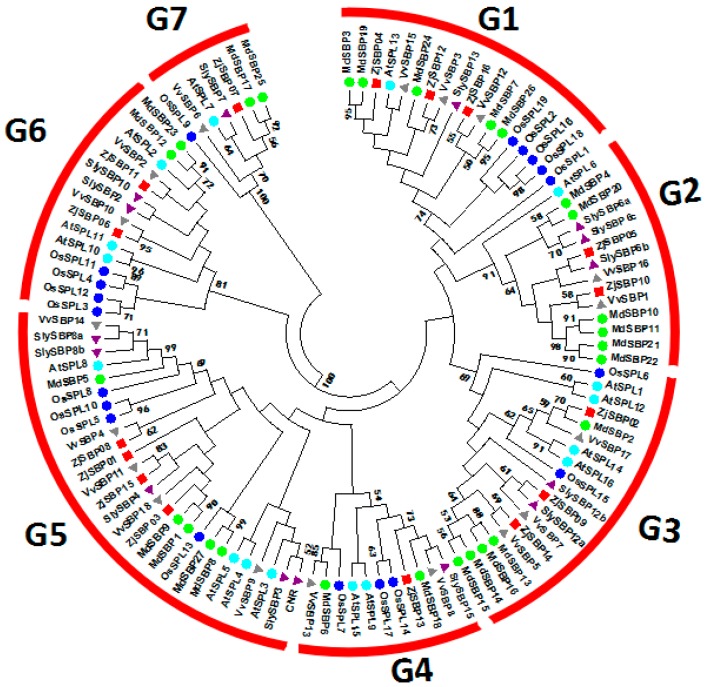
Phylogenetic analysis of jujube and other SBP-box families by MEGA 7.0. The sequences of the SBP domain are from *Arabidopsis* (AtSPL), apple (MdSBP), rice (OsSPL), tomato (SlySBP or CNR), grape (VvSBP) and jujube (ZjSBP). The sequences and the sources of other plants are in Tables S2 and S3.

In order to further understand the evolutionary relationship of the ZjSBP genes and help to reveal roles of these ZjSBPs in jujube development, we employed 111 putative SBP sequences from six species, including monocotyledonous angiosperms (rice) and five dicotyledonous angiosperms (*Arabidopsis*, apple, grape, tomato and jujube) to construct a phylogenetic tree using MEGA 7.0 [29] (Figure 3). Only the conserved SBP domain (74 aa) were used for the phylogenetic tree construction (Table S1). These 111 sequences were classified into seven groups. Interestingly, all the OsSPL genes in each group were very dissimilar to other dicotyledons and most of the ZjSBP genes were closer to grape, apple, tomato and *Arabidopsis* than rice. The ZjSBP genes were distributed in all the groups.

### 2.3. Gene Structures of the ZjSBP Genes

**Figure 4:**
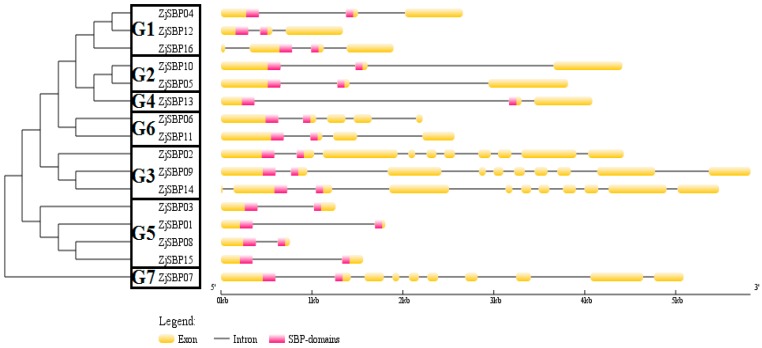
Phylogenetic analysis of jujube SBP domain proteins and exon-intron structures with the online Gene Structure Display Server. Exons and SBP domains are indicated by yellow and pink boxes. Black lines connecting two exons represent introns.

To gain further insight into the structures of the ZjSBP genes, we generated the exon-intron structures based on their corresponding genome sequences and coding sequences (Figure 4). The groups corresponded to the phylogenetic groups discussed above. All members had an intron at the highly conserved position in the SBP-box [30]. All the ZjSBP genes had a similar structure of exon-intron within the same group, while the number of exons varied among groups. For example, the genes in Group 5 had two exons, whereas ZjSBP14 in Group 3 had eleven exons. Furthermore, except for ZjSBP14 and ZjSBP16, the SBP domains of all genes were distributed in the first and second exons.

**Figure 5:**
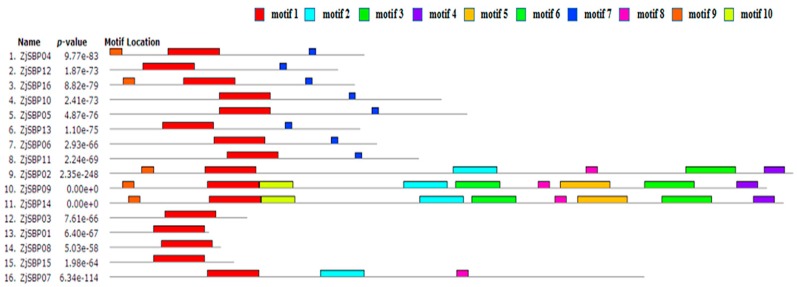
The motifs of each *ZjSBP* gene by MEME. Numbers 1–10 are displayed in different colored boxes. The sequence information for each motif is provided in Table 2.

We searched for the presence of any conserved motifs in the ZjSBP genes using the MEME software (Figure 5). Combined with the results of the InterProScan, Motif 1 consisted of the SBP domain and Motif 3 was the Ankyrin repeat-containing domain. Other motifs were unknown for their functions (Table 2). Furthermore, some motifs—for example Motif 3 and Motif 5—were only found in one unique group but were shared by all the members within the group. The best possible matches are shown in Table 3.

### 2.4. Homology Analysis

**Figure 6:**
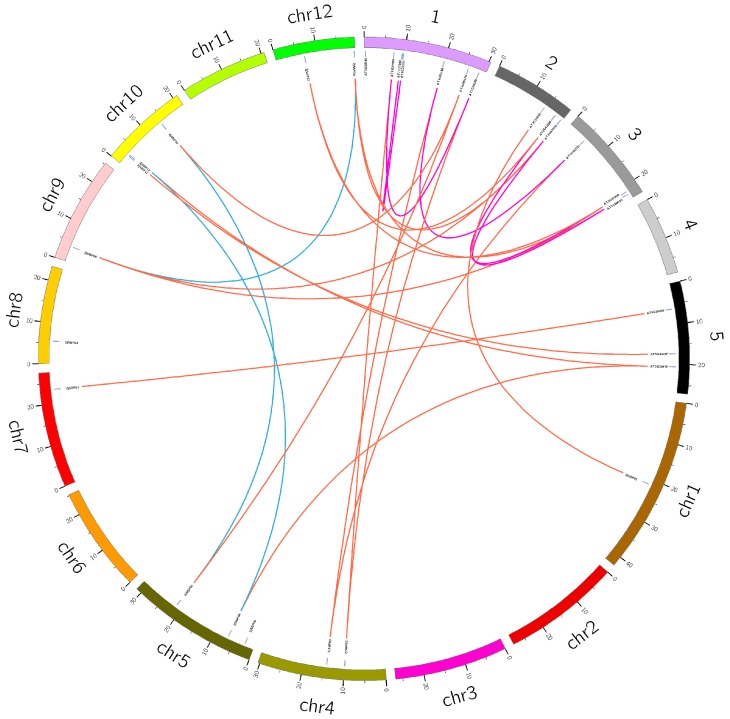
The homologous gene pairs between Z. *jujuba* Mill. Dongzao and *Arabidopsis* with Circus. Red, purple and blue lines indicate homologous gene pairs between Z. *jujuba* Mill. Dongzao and *Arabidopsis*, and *Arabidopsis* and Z. *jujuba* Mill. Dongzao.

The members located on the scaffold were excluded because the length was too short to draw a picture of the results. According to OrthoMCL, there were 12 homologous gene pairs between jujube and *Arabidopsis*, five gene pairs in *Arabidopsis* and three gene pairs in jujube. The result was visualized using the Circos (Version 0.69) [31] (Table S1, Figure 6).

### 2.5. Expression Analysis of the ZjSBP Genes

**Figure 7:**
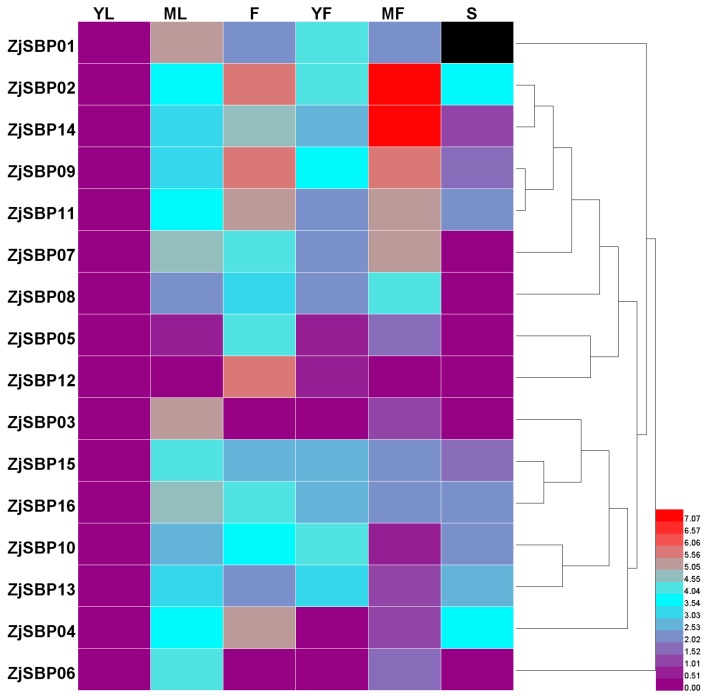
Expression levels of ZjSBPs in different tissues. Differences in gene expression are shown in color according to the scale by Heml 1.0. Tissues or organs: YL—young leaves; ML—mature leaves; F—flowers; YF—young fruits; MF—mature fruits; S—shoots.

To further confirm the function of ZjSBPs during jujube vegetative and reproductive growth, we explored the expression patterns of each gene by qRT-PCR. Generally, the expression profiles of ZjSBPs can be divided into two types (Figure 7). One type is similar to ZjSBP08, ZjSBP10, ZjSBP13, ZjSBP15 and ZjSBP16; they seemed to be expressed constitutively from leaves to fruits and their expression levels were similar between each tissue. Another type relates to genes whose expression levels in some tissues were clearly different, such as ZjSBP02 in mature fruits, ZjSBP12 in flowers and ZjSBP14 in mature fruits. The expression of ZjSBP01 was not detected in the stems. Among all ZjSBPs, ZjSBP02 and ZjSBP14 clearly expressed high levels in mature fruits. ZjSBP01 showed higher expression in mature leaves than other tissues. The expression levels of ZjSBP12 was much higher in flowers than other tissues.

### 2.6. Expression Patterns of the Transcriptome of Different Tissues

**Figure 8:**
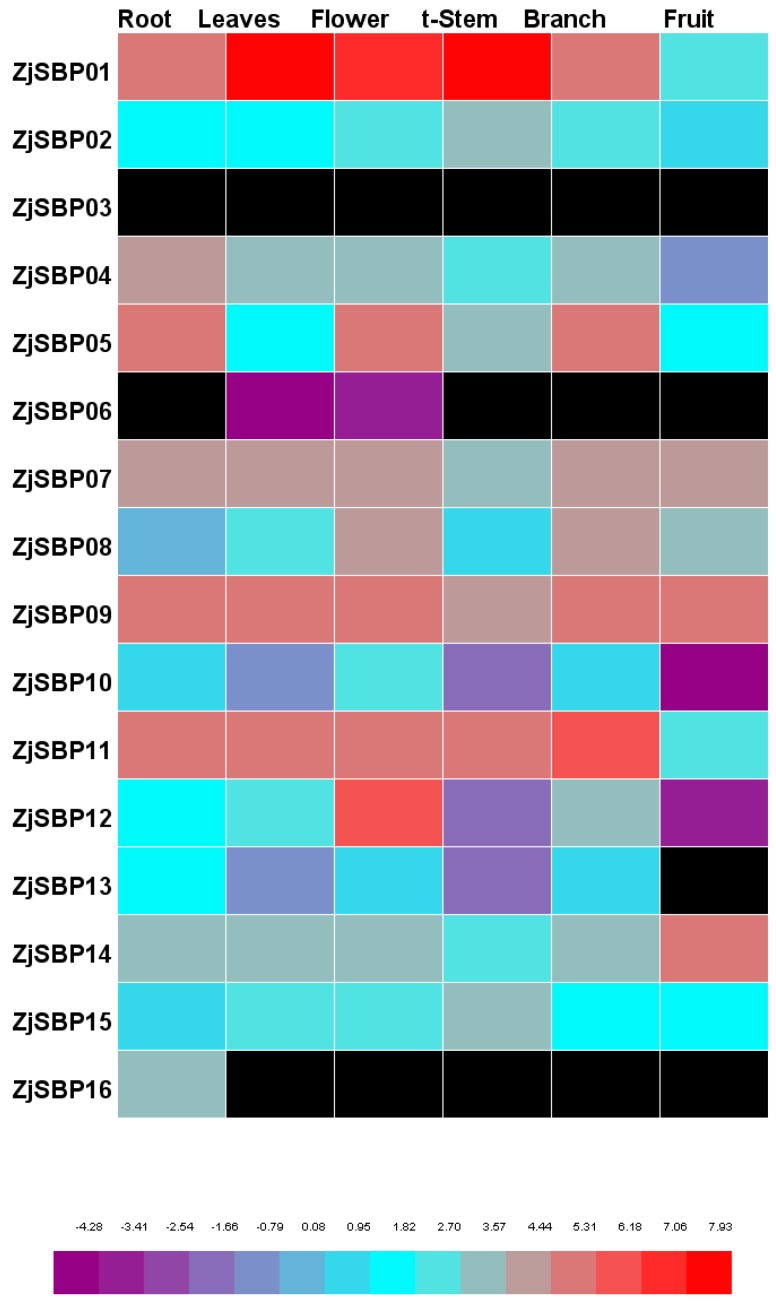
Expression quantities of ZjSBPs for different transcriptomes in different tissues by Heml 1.0.

The expression levels of each of the genes in different tissues are shown in Figure 8. According to the FPKM value, most genes expressed clearly in all tissues except for ZjSBP03, ZjSBP06, ZjSBP13 and ZjSBP16. ZjSBP01 reached clearly higher levels in s-stem and leaves. ZjSBP06 only expressed in leaves and flowers and ZjSBP16 only expressed in the root. The expression value of ZjSBP13 was zero in fruit. The transcript of ZjSBP16 was zero in all tissues samples.

## 3. Discussion

SBP-box gene family encodes the transcription factors which are plant specific. At first, we identified 37 sequences from jujube. LOC107424208 was excluded for its uncompleted SBP domain. LOC107408877, LOC107408879, LOC107408885, LOC107409175 and LOC107409984 was similar to LOC107408850, so we chose LOC107408850 to represent these genes. LOC107404563 was similar to LOC107416051, LOC107432574 was similar to LOC10741647 and LOC107422456 was similar to LOC10741647 (only one amino acid was different, maybe due to a fault in the chromosome package of Junzao) [24]. We therefore chose LOC107416051 to represent LOC107404563 and LOC10741647 to represent LOC107432574 and LOC107422456, respectively. The different functions of these similar sequences can be explored in the future.

According to the phylogenetic analysis, all members from jujube are close to the apple, grape and tomato. Compared with rice, the ZjSBP genes were clustered more tightly with dicotyledon, which coincides with the fact that they diverged more recently from a common ancestor, rather than the lineage which produced the monocotyledon. These results indicate that although the SBP-box genes may come from the same ancestor and exist after the divergence of plants and animals, they have different differentiating patterns after each lineage was separated.

The differences of each group are reflected in the length of each gene. The amino acids length in Group 5, which contained ZjSBP01, ZjSBP03, ZjSBP08 and ZjSBP15 was approximately two hundred aa, but in Group 3 there were about one thousand aa for each member (Table 1). Intriguingly, the gene structures of different groups were also obvious. Genes in Group 2 had three exons (Figure 4) but genes in Group 7 had ten exons. For motifs, all members in Group 5 only had the SBP domain-motif 1, nevertheless, there were more than six motifs in Group 3 (Figure 5). Within the same phylogenetic group, members mostly had a similar exon-intron structure and motif structure, indicating that the evolution of the SBP-box gene family may be closely related to the diversification of gene structures, as explained before in rice [6].

The results of the quantitative RT-PCR indicated that ZjSBPs had different expressed patterns in various organs and at different stages of leaf and fruit development. The transcriptome data also showed that ZjSBPs had different expression patterns in different tissues (Figure 8). According to the transcriptome data, the expression of ZjSBP03 was zero and ZjSBP06 only expressed weakly in leaves and flowers. At the same time, the result of qRT-PCR indicated that ZjSBP03 and ZjSBP06 expressed very low in all tissues except for mature leaves. ZjSBP13 and ZjSBP15 exhibited low-level, constitutive expression in all tissues both from qRT-PCR and the transcriptome data. Interestingly, there were some discrepancies between the results of qRT-PCR and the transcriptome data. In terms of qRT-PCR, ZjSBP02 and ZjSBP14 exhibited high expression levels in mature fruit. However, the expression levels of ZjSBP02 and ZjSBP14 were weak in fruit according to the transcriptome data. Unfortunately, we did not have the information for the exact development stages of fruit employed in the RNA-seq data and the difference may be due to the fruits being from different development stages.

The functions of SBP-box genes have been identified to play vital roles in regulating flower and fruit development and other physiological processes, but in jujube, the functions are not clear. We predicted the functions of ZjSBPs based on their evolution relationships and the homology of other SBP-box genes. All members of ZjSBPs in Group 1 exhibited different expression profiles but have high expression levels in leaves and flowers. They were close to AtSPL13 in the phylogenetic tree, which was expressed mainly in the hypocotyl and affected leaf primordium development [32]. Interestingly, the homology analysis revealed that ZjSBP04 and ZjSBP12 were homologous with AtSPL13. This indicates that these genes may have a similar function in leaf or shoot development. There is an orthologous gene of AtSPL13 in maize named tga1 (teosinte glume architecture 1), which has been found to be involved in ear glume development in maize [33]. Furthermore, it has been confirmed that the OsSPL16 in this group influences grain size, shape and quality [16].

There were few reports about the genes from Group 2. The ZjSBPs in this group expressed a little high in flowers and young fruits. ZjSBP05 and ZjSBP10 may have functions during these tissues’ development. The genes in Group 3 are very highly expressed in mature fruits, almost ten times that of the young fruits, indicating that these genes may be related to the later stages of fruit development. Group 3 is close to AtSPL14, which has been found to relate to programmed cell death in response to the fungal toxin fumonisin B1 [34]. The homology analysis also showed that ZjSBP02 was homologous with AtSPL14. We can infer that these three ZjSBPs may have a similar function during plant development. In Group 4, ZjSBP13 expressed similarly in each tissue. We can find in the phylogenetic tree that AtSPL9, AtSPL15 and OsSPL14 were in this group. Meanwhile, AtSPL9 and AtSPL15 with ZjSBP13 were the homologous gene pair. The function of AtSPL9 and AtSPL15 was to control the shoot maturation and act redundantly in controlling the juvenile-to-adult phase transition [15]. Moreover, AtSPL9 plays an important role during the flowering and negatively regulates the accumulation of anthocyanin [35,36]. OsSPL14 has been proven to influence the panicle development and affect grain productivity [15]. ZjSBP07 is the only jujube member in Group 7. The homologous gene in *Arabidopsis* is AtSPL7, which has been shown to replay the copper deficiency [37].

In Group 5, CNR has been found to be critical for normal ripening in the tomato [18]. AtSPL8 was clustered in this group. Some reports found that AtSPL8 played a significant role in male fertility and anther development [9,38]. ZjSBP06 and ZjSBP11 are in Group 6, which contains AtSPL2, AtSPL10 and AtSPL11. Notably, AtSPL2 was the homologous gene with ZjSBP11. These three genes have been reported to control the development of lateral organs in association with shoot maturation in the reproductive phase [39].

## 4. Materials and Methods

### 4.1. Plant Material

Young leaves (the second to fifth fully expanded young leaves under shoot apices when new shoots were 40–60 cm in length), mature leaves (leaves at the bottom of new shoots with a length of 40–60 cm), shoots, flowers, young fruit on July 13 (green fruit about 1 cm in diameter) and mature fruit on September 3 (ripe fruit about 3 cm in diameter) of *Z. jujuba* Mill. Dongzao (Dongzao) were sampled from the National Key Base for Improved Chinese Jujube Cultivar (Cangzhou, China). All the plant tissues were immediately frozen in liquid nitrogen and stored at −80 °C for later research.

### 4.2. Identification of SBP-Box Genes in Dongzao

The protein sequence of the SBP-box gene family in *Arabidopsis* were downloaded from the *Arabidopsis* Transcription Factors Database website (http://datf.cbi.pku.edu.cn/). Subsequently, these sequences were used to search the Dongzao genome (version 1.1) at the NCBI (http://blast.ncbi.nlm.nih.gov/Blast.cgi) using blast with a cut-off E-value of 1 × 10^−5^. We reorganized and merged the highly matched sequences and used InterProScan (http://www.ebi.ac.uk/Tools/pfa/iprscan5/) to scan the protein domain. The EST database in NCBI was used to further check the sequence of this gene family.

### 4.3. Sequence Alignments, Phylogenetic Analyses and Exon-Intron Structure Determination

We used ExPASy (http://www.expasy.org/) to compute the physical and chemical parameters of each protein sequence. Multiple sequence alignment was carried out on DNAMAN software (Version 6.0, Lynnon Biosoft, Quebec, QC, Canada). The sequence logo was obtained using the online Weblogo platform (http://weblogo.berkeley.edu). Phylogenetic trees were constructed using MEGA 7.0 software with the neighbor-joining (NJ) method and the bootstrap test replicated 1000 times. We aligned the coding sequences to their corresponding genomic sequences to get the exon-intron structures of the SBP-box genes. The graph of the exon-intron structures was obtained with the online Gene Structure Display Server (GSDS: http://gsds.cbi.pku.edu.ch). Furthermore, MEME (http://meme-suite.org/) was used to search for motifs in all SBP-box genes. The number of motifs was set to 10.

### 4.4. Homology Analysis

OrthoMCL [40] was used to search for orthologous and paralogous genes in jujube and *Arabidopsis* using the entire protein sequences. The results of the OrthoMCL were displayed by Circos [30].

### 4.5. Expression Analysis of the ZjSBP Genes

Beacon Designer 7.9 was used to design the gene-specific primers of each SBP-box gene (Table S2). All primers were designed avoiding the conserved domains. These sequences were subsequently verified using the BLAST tool at NCBI and a dissociation curve was also analyzed after the PCR reaction to confirm their specificity. The jujube actin gene (EU916201) was used as an internal control to normalize the expression level of the target genes among different samples. The total RNA of different tissues was extracted by the E.Z.N.A.TM Plant RNA Kit (OMEGA). Residual DNA was removed through treatment with DNase I (Promega, Madison, WI, USA). The quantity of the total RNA was detected by NanoDrop 2000 (Thermo Fisher Scientific, Waltham, MA, USA). One microgramme of the total RNA was used for first-strand cDNA synthesis with a mixture of Poly dT and random hexamer primers (PrimeScriptTM RTase, TaKaRa Biotechnology, Dalian, China). The products were diluted ten times to be templates for later qRT-PCR, and stored at −20 °C. Quantitative RT-PCR was performed in the presence of SYBR Green (TaKaRa Biotechnology) and read on an IQ5 real-time PCR instrument (Bio-Rad, Hercules, CA, USA). Each reaction was performed in triplicate with a volume of 10 μL. The following program was used for qRT-PCR: 95 °C for 3 min followed by 39 cycles at 95 °C for 20 s, 55 °C for 20 s, 72 °C for 20 s.

### 4.6. Expression Quantity of the Transcriptome of Different Tissues

The data of transcriptomes from six tissues (the root, leaves, flower, t-stem, branch and fruit) of Dongzao were retrieved from NCBI with accession number PRJNA260241, which was reported previously by Liu et al. 2014 [28]. Raw sequences were filtered to remove adaptor-containing readings, readings with more than 10% unknown nucleotides, and low-quality readings with more than 50% of bases with a quality value ≤5. Evaluation of the quality of RNA-seq readings and trimming of low-quality readings (Phred quality (Q) score < 20) were carried out using FastQC v0.11.5. The readings were mapped to the jujube genome using TopHat2 version 2.1.1 [41] with default parameters. The Cufflinks Version 2.2.1 with default parameters was employed to perform the quantitative analysis of gene expression based on FPKM-values (Fragments Per Kilobase of gene model per Million fragments mapped) [42]. The different expression profiles were demonstrated by heat map with Heml 1.0 software (CUCKOO, China).

## 5. Conclusions

The SBP-box gene family is specific to plants and encodes transcription factors with a wide range of functions. To date, the SBP-box gene family of many species has been investigated but there is little information concerning jujube. In the present study, we identified 16 SBP-box gene family members in jujube. Subsequently, gene structures and exon-intron were examined. The phylogenetic analysis and homology analysis indicated the relationship between jujube and other species, from which we can conjecture the corresponding functions. Most ZjSBPs may play vital roles in flower, leaf and fruit development. Expression patterns in different tissues also revealed the potential functions. The results indicated fundamental information about ZjSBPs, which would provide valuable data to further investigate ZjSBP genes in jujube.

## Figures and Tables

**Table 1:** The SBP-box gene family in Z. *jujuba* Mill. Dongzao.

Gene Name	Gene ID	RefSeq ID	Location	CDS (bp)	Amino Acids Length (aa)	MW (kDa)	pI	GRAVY	Instability Index (II)	EST
ZjSBP01	LOC107425063	XM_016034996.1	chr01:21341210-21343540	465	154	17,411.90	6.66	−1.382	88.82	YES
ZjSBP02	LOC107416051	XM_016024498.1	chr04:9923829-9929510	3180	1059	117,174.39	8.73	−0.476	60.91	YES
ZjSBP03	LOC107416470	XM_016024954.1	chr04:14303210-14305417	642	213	23,753.22	9.04	−1.127	60.78	NO
ZjSBP04A	LOC107418038	XM_016026704.1	chr05:6738154-6742177	1185	394	42,994.94	7.26	−0.576	53.17	YES
ZjSBP04B	LOC107418038	XM_016026705.1	chr05:6738154-6742177	1185	394	42,994.94	7.26	−0.576	53.17	YES
ZjSBP05	LOC107419077	XM_016027806.1	chr05:21507138-21511275	1665	554	60,692.96	8.07	−0.487	51.59	YES
ZjSBP06	LOC107417566	XM_016026174.1	chr05:2335756-2337973	1245	414	45,301.32	8.54	−0.580	58.86	NO
ZjSBP07A	LOC107423307	XM_016032840.1	chr07:23461384-23466777	2487	828	92,658.80	6.20	−0.427	56.10	YES
ZjSBP07B	LOC107423307	XM_016032841.1	chr07:23461384-23466777	2400	799	89,422.84	6.04	−0.481	55.73	YES
ZjSBP08	LOC107424290	XM_016034051.1	chr08:5519236-5520446	519	172	19,646.28	5.20	−1.309	78.87	YES
ZjSBP09A	LOC107426205	XM_016036321.1	chr09:3743974-3750726	3057	1018	112,964.47	6.22	−0.482	49.87	YES
ZjSBP09B	LOC107426205	XM_016036322.1	chr09:3743974-3750726	3057	1018	112,964.47	6.22	−0.482	49.87	YES
ZjSBP09C	LOC107426205	XM_016036323.1	chr09:3743974-3750726	3057	1018	112,964.47	6.22	−0.482	49.87	YES
ZjSBP10	LOC107429070	XM_016039720.1	chr10:13935247-13941596	1545	514	57,356.09	7.56	−0.610	56.15	YES
ZjSBP11A	LOC107428311	XM_016038829.1	chr10:2775470-2780707	1440	479	52,124.50	7.24	−0.762	56.96	YES
ZjSBP11B	LOC107428311	XM_016038830.1	chr10:2775470-2780707	1440	479	52,124.50	7.24	−0.762	56.96	YES
ZjSBP11C	LOC107428311	XM_016038831.1	chr10:2775470-2780707	1440	479	52,124.50	7.24	−0.762	56.96	YES
ZjSBP11D	LOC107428311	XM_016038833.1	chr10:2775470-2780707	1440	479	52,124.50	7.24	−0.762	56.96	YES
ZjSBP12A	LOC107428287	XM_016038800.1	chr10:2934047-2937123	1065	354	39,239.78	8.54	−0.723	63.10	YES
ZjSBP12B	LOC107428287	XM_016038801.1	chr10:2934047-2937123	1032	343	37,911.27	8.55	−0.717	62.37	YES
ZjSBP13A	LOC107432336	XM_016043455.1	chr12:6956478-6961330	1167	388	41,547.47	9.11	−0.532	54.74	YES
ZjSBP13B	LOC107432336	XM_016043456.1	chr12:6956478-6961330	1146	381	40,762.53	9.11	−0.542	55.78	YES
ZjSBP14	LOC107433357	XM_016044643.1	chr12:19190329-19195805	3135	1044	116,394.53	7.84	−0.419	50.17	YES
ZjSBP15	LOC107406208	XM_016013312.1	add_scaffold 414:32884-34897	579	192	21,583.47	9.56	−0.992	66.24	YES
ZjSBP16A	LOC107408850	XM_016016273.1	add_scaffold2409:1682-4023	1167	388	43,549.82	8.29	−0.743	53.37	YES
ZjSBP16B	LOC107408850	XM_016016274.1	add_scaffold2409:1682-4023	1143	380	47,233.32	8.98	−0.757	52.77	YES
ZjSBP16C	LOC107408850	XM_016016275.1	add_scaffold2409:1682-4023	1083	360	40,305.19	8.490	−0.751	57.42	YES
ZjSBP16D	LOC107408850	XM_016016277.1	add_scaffold2409:1682-4023	1083	360	40,305.19	8.490	−0.751	57.42	YES

**Table 2:** Analysis and distribution of conserved motifs in ZjSBPs.

Motif No.	E-Value	Sites	Width	Annotation of Motif
1	1.7 × 10^−69^	16	80	SBP domain
2	3.3 × 10^−67^	4	68	unknown
3	6.3 × 10^−36^	3	78	Ankyrin repeat
4	1.6 × 10^−17^	3	33	unknown
5	2.3 × 10^−16^	2	78	unknown
6	1.1 × 10^−9^	2	70	unknown
7	1.6 × 10^−7^	8	11	unknown
8	1.7 × 10^−7^	4	19	unknown
9	2.1 × 10^−5^	5	19	unknown
10	1.2 × 10^−4^	2	53	unknown

**Table 3:** Best possible match of conserved motifs.

Name	E-Value	Best Possible Match
Motif 1	1.7 × 10^−69^	PSCQVEGCNADLSSAKDYHRRHKVCELHSKAPKVJVGGLEQRFCQQCSRFHELSEFDEGKRSCRRRLAGHNERRRKPQPE
Motif 2	3.37 × 10^−67^	AQSRTGRIVFKLFGKDPNDFPLVLRAQILDWLSNSPSDIESYIRPGCIILTIYLAMPEAAWEELCENL
Motif 3	6.37 × 10^−36^	AQSRTGRIVFKLFGKDPNDFPLVLRAQILDWLSNSPSDIESYIRPGCIILTIYLAMPEAAWEELCENL
Motif 4	1.67 × 10^−17^	LLYRPAMLSMVAIAAVCVCVALLFKSSPEVVYV
Motif 5	2.37 × 10^−16^	GNIEAKKQALDFIHEMGWLLHRSRAKLRLGHLDPNADPFPFKRFKWLMEFSLEHDWCAVVKKLLGILFEGSVDEGEHP
Motif 6	1.17 × 10^−9^	DPFWRTGWVYIRLQNFIAFIYNGHVIJDTPLPLKSHKNCKILSIKPIAISASEKAQFIVKGFNLARPATR
Motif 7	1.67 × 10^−7^	CALSLLSSQPT
Motif 8	1.77 × 10^−7^	FFPFIVADEEVCSEIRVLE
Motif 9	2.17 × 10^−5^	LEWDLKDWSWDGTLFLAEP
Motif 10	1.27 × 10^−4^	TVVNGNSLNDERGSGYLLISLLRILSNMHSNRSDQNKDQDLLSHLLRSLANFT

## Supplementary Materials

Supplementary materials can be found at www.mdpi.com/1422-0067/18/8/1734/s1.
