# Supplementary material for: Genome-Wide Organization and Expression Profiling of the SBP-Box Gene Family in Chinese Jujube (Ziziphus jujuba Mill.)
Source: Int J Mol Sci. 2017 Aug 15;18(8):1734. doi: 10.3390/ijms18081734 (PMC5578124; doi:10.3390/ijms18081734)
Supplement: Supplementary file 1 [file ijms-18-01734-s001.pdf]

Supplementary Table 1 General information of SBP-box genes selected for phylogenetic analysis.

Gene	Accession no. or locus ID	Protein sequence of SBP-domain
AtSPL1	At2g47070	CQVENCEADLSKVKDYHRRHKVCEMHSKATSATVGGILQRFCQQCSRFHLLQEFDEGKRSCRRRLAGHNKRRRK
AtSPL2	At5g43270	CQVEGCNLDLSSAKDYHRKHRICENHSKFPKVVVSGVERRFCQQCSRFHCLSEFDEKKRSCRRRLSDHNARRRK
AtSPL3	At2g33810	CQVESCTADMSKAKQYHKRHKVCQFHAKAPHVRISGLHQRFCQQCSRFHALSEFDEAKRSCRRRLAGHNERRRK
AtSPL4	At1g53160	CQVDRCTADMKEAKLYHRRHKVCEVHAKASSVFLSGLNQRFCQQCSRFHDLQEFDEAKRSCRRRLAGHNERRRK
AtSPL5	At3g15270	CQVDRCTVNLTEAKQYYRRHRVCEVHAKASAATVAGVRQRFCQQCSRFHELPEFDEAKRSCRRRLAGHNERRRK
AtSPL6	At1g69170	CQVYGCSKDLSSSKDYHKRHRVCEAHSKTSVVIVNGLEQRFCQQCSRFHFLSEFDDGKRSCRRRLAGHNERRRK
AtSPL7	At5g18830	CQVPDCEADISELKGYHKRHRVCLRCATASFVVLDGENKRYCQQCGKFHLLPDFDEGKRSCRRKLERHNNRRKR
AtSPL8	At1g02065	CQAEGCNADLSHAKHYHRRHKVCEFHSKASTVVAAGLSQRFCQQCSRFHLLSEFDNGKRSCRKRLADHNRRRRK
AtSPL9	At2g42200	CQVEGCGMDLTNAKGYYSRHRVCGVHSKTPKVTVAGIEQRFCQQCSRFHQLPEFDLEKRSCRRRLAGHNERRRK
AtSPL10	At1g27370	CQIDGCELDLSSSKDYHRKHRVCETHSKCPKVVVSGLERRFCQQCSRFHAVSEFDEKKRSCRKRLSHHNARRRK
AtSPL11	At1g27360	CQIDGCELDLSSAKGYHRKHKVCEKHSKCPKVSVSGLERRFCQQCSRFHAVSEFDEKKRSCRKRLSHHNARRRK
AtSPL12	At3g60030	CQVDNCGADLSKVKDYHRRHKVCEIHSKATTALVGGIMQRFCQQCSRFHVLEEFDEGKRSCRRRLAGHNKRRRK
AtSPL13	At5g50570	CLVDGCDSDFSNCREYHKRHKVCDVHSKTPVVTINGHKQRFCQQCSRFHALEEFDEGKRSCRKRLDGHNRRRRK
AtSPL14	At1g20980	CQVDNCTEDLSHAKDYHRRHKVCEVHSKATKALVGKQMQRFCQQCSRFHLLSEFDEGKRSCRRRLAGHNRRRRK
AtSPL15	At3g57920	CQVEGCRMDLSNVKAYYSRHKVCCIHSKSSKVIVSGLHQRFCQQCSRFHQLSEFDLEKRSCRRRLACHNERRRK
AtSPL16	At1g76580	CQVDNCKEDLSIAKDYHRRHKVCEVHSKATKALVGKQMQRFCQQCSRFHLLSEFDEGKRSCRRRLDGHNRRRRK

MdSBP1	MDP0000861601 or MDP0000156994	CQVERCGADLVDAKRYYRRHKVCEFHSKAAVVIVSGIQQRFCQQCSRFHELIEFDEAKRSCRRRLAGHNERRRK
MdSBP2	MDP0000149339	CQVDNCKEDLSNAKDYHRRHKVCEVHSKSTRALVAKQMQRFCQQCSRFHPLSEFDEGKRSCRRRLAGHNRRRRK
MdSBP3	MDP0000263766	CLVDGCNSDLSTCRDYHRRHKVCELHSKTPQVTINGNKQRFCQQCSRFHAPEEFDEGKRSCRKRLDGHNRRRRK
MdSBP4	MDP0000146640 or MDP0000778465	CQVYGCNKDLSSCKDYHKRHKVCEVHSKTAKVIINGIEQRFCQQCSRFHLLGEFDDGKRSCRKRLAGHNERRRK
MdSBP5	MDP0000803116	CQAEGCAADLSHSKHYHRRHKVCEFHSKASTVIANGLTQRFCQQCSRFHLLSEFDNGKRSCRKRLADHNRRRRK
MdSBP6	MDP0000262141	CQVEGCHVALLNAKEYHRRHKVCAMHSKASRVTVLGQDQRFCQQCSRFHVVSEFDESKRSCRRRLAGHNERRRK
MdSBP7	MDP0000170630	CLVDGCRADLSRCREYHRRHRVCELHSKAPVVVVRGEQKRFCQQCSRFHSLVDFDKVKRSCRKRLNGHNQRRRK
MdSBP8	MDP0000865739	CQADRCTADLSEEKQYHRKHKVCDLHSKSQVVLVSGLHQRFCQQCSRFHQLPEFDDTKRSCRRRLAGHNERRRK
MdSBP9	MDP0000158607	CQAERCGADLVDAKRYHRRHKVCEFHSKAAVVIVSGTRQRFCQQCSRFHELIEFDEAKRSCRRRLAGHNERRRK
MdSBP10	MDP0000171877	CQVHGCNMDLTFSKDYHKRHRVCDAHSKTAVVIVNGIKQRFCQQCSRFHLLAEFDDVKRSCRRRLAGHNLRRRK
MdSBP11	MDP0000246046	CQVHGCNMDLTFSKDYHKRHRVCDAHSKTAVVIVNGIKQRFCQQCSRFHLLAEFDDVKRSCRRRLAGHNLRRRK
MdSBP12	MDP0000155354	CQVEGCNLDLSSVKDYHRKHRICANHSKSPKVVVDGVERRFCQQCSRFHGLSEFDENKRSCRRRLSDHNARRRK
MdSBP13	MDP0000919693	CQVEDCKADLSNAKDYHRRHKVCDMHSKATKAVVGNVLQRFCQQCSRFHGLQEFDEGKRSCRRRLAGHNRRRRK
MdSBP14	MDP0000919694	CQVEDCKADLSNAKDYHRRHKVCAMHSKATKALVGSVMQRFCQQCSRFHALQEFDEGKRSCRRRLAGHNRRRRK
MdSBP15	MDP0000180408	CQVEDCKADLSNAKDYHRRHKVCDMHSKATKALVGNVMQRFCQQCSRFHALQEFDEGKRSCRRRLAGHNRRRRK
MdSBP16	MDP0000180409	CQVEDCKADLSNAKDYHRRHKVCDMHSKATKAPVGNVLQRFCQQCSRFHVLQEFDEGRRSCRRRLAGHNRRRRK
MdSBP17	MDP0000271587	CQVPSCGIDIKELKGYHRRHRVCLACANAITVVIDGETKRYCQQCGKFHVLPDFDEGKRSCRRKLERHNNRRRR

MdSBP18	MDP0000297978	CQVEGCQVDLSDAKAYYSRHKVCGLHSKTPTVIVAGLEQRFCQQCSRFHLLPEFDQGKRSCRRRLAGHNERRRK
MdSBP19	MDP0000210138	CLVDGCNCDLSTCRDYHRRHKVCELHSKTPQVTINGNKQRFCQQCSRFHALEEFDQGKRSCRKRLDGHNRRRRK
MdSBP20	MDP0000589558	CQVYGCNKDLSSYKDYHKRHKVCEVHSKTAKVIVNGIEQRFCQQCSRFHLLGEFDDGKRSCRRRLAGHNERRRK
MdSBP21	MDP0000193702	CQVHGCNMDLTFSKDYHKRHRVCDAHSKTAVVIVNGIEQRFCQQCSRFHLLAEFDDVKRSCRKRLAGHNLRRRK
MdSBP22	MDP0000119458	CQVHGCNMDLTFSKDYHKRHRVCDAHSKTAVVIVNGIEQRFCQQCSRFHLLAEFDDVKRSCRKRLAGHNLRRRK
MdSBP23	MDP0000249364	CQVEGCNLDLSSAKDYHRKHRICSNHSKSPKVVVDGVELRFCQQCSRFHGLSDFDENKRSCRKRLSDHNARRRK
MdSBP24	MDP0000176265	CLVDGCNSDLSKCRDYHRRHKVCELHSKTSKVTIKGQERRFCQQCSRFHSLVEFDEGKRSCRRRLDGHNKRRRK
MdSBP25	MDP0000195461	CQVPSCGVDIKELKGYHRRHRVCLACANASTVDIDGESKRYCQQCGKFHVLSDFDEGKRSCRRKLERHNNRRRR
MdSBP26	MDP0000142582	CLVDDCRADLSRCREYHRRHRVCELHSKTPVVVVKGEQKRFCQQCSRVHSLVEFDDGKKSCRKRLNGHNQRRRK
MdSBP27	MDP0000255162 or MDP0000538106	CQADRCTADLSDEKKYHRKHKVCDLHSKSQVVLVSGLHQRFCQQCSRFHEXSEFDDTKRSCRSRLSGHNERRRK
OsSPL1	LOC_Os01g18850.1	CQVDGCTVNLSSARDYNKRHKVCEVHTKSGVVRIKNVEHRFCQQCSRFHFLQEFDEGKKSCRSRLAQHNRRRRK
OsSPL2	LOC_Os01g69830.1	CSVEGCAADLSKCRDYHRRHKVCEAHSKTAVVTVAGQQQRFCQQCSRFHLLGEFDEEKRSCRKRLDGHNKRRRK
OsSPL3	LOC_Os02g04680.1	CQVEGCNVDLSSAKPYHRKHRVCEPHSKTLKVIVAGLERRFCQQCSRFHGLAEFDQKKRSCRRRLHDHNARRRK
OsSPL4	LOC_Os02g07780.1	CQVEGCGVELVGVKDYHRKHRVCEAHSKFPRVVVAGQERRFCQQCSRFHALSEFDQKKRSCRRRLYDHNARRRK
OsSPL5	LOC_Os02g08070.1	CQAEGCKADLSAAKHYHRRHKVCDFHAKAAAVLAAGKQQRFCQQCSRFHVLAEFDEAKRSCRKRLTEHNRRRRK
OsSPL6	LOC_Os03g61760.1	CQVEGCTADLTGVRDYHRRHKVCEMHAKATTAVVGNTVQRFCQQCSRFHPLQEFDEGKRSCRRRLAGHNRRRRK
OsSPL7	LOC_Os04g46580.1	CQVEGCDITLQGVKEYHRRHKVCEVHAKAPRVVVHGTEQRFCQQCSRFHVLAEFDDAKKSCRRRLAGHNERRRR
OsSPL8	LOC_Os04g56170.1	CQAEGCKADLSSAKRYHRRHKVCEHHSKAPVVVTAGLHQRFCQQCSRFHLLDEFDDAKKSCRKRLADHNRRRRK

OsSPL9	LOC_Os05g33810.1	CQVPGCEADIRELKGYHRRHRVCLRCAHAAAVMLDGVQKRYCQQCGKFHILLDFDEDKRSCRRKLERHNRRRRK
OsSPL10	LOC_Os06g44860.1	CQAEGCKADLSGAKHYHRRHKVCEYHAKASVVAASGKQQRFCQQCSRFHVLTEFDEAKRSCRKRLAEHNRRRRK
OsSPL11	LOC_Os06g45310.1	CQVEGCGLELGGYKEYYRKHRVCEPHTKCLRVVVAGQDRRFCQQCSRFHAPSEFDQEKRSCRRRLSDHNARRRK
OsSPL12	LOC_Os06g49010.1	CQVEGCKVDLSSAREYHRKHKVCEAHSKAPKVIVSGLERRFCQQCSRFHGLAEFDQKKKSCRRRLSDHNARRRK
OsSPL13	LOC_Os07g32170.1	CQVERCGVDLSEAGRYNRRHKVCQTHSKEPVVLVAGLRQRFCQQCSRFHELTEFDDAKRSCRRRLAGHNERRRK
OsSPL14	LOC_Os08g39890.1	CQVEGCGADLSGIKNYYCRHKVCFMHSKAPRVVVAGLEQRFCQQCSRFHLLPEFDQGKRSCRRRLAGHNERRRR
OsSPL15	LOC_Os08g40260.1	CQVDDCRADLTNAKDYHRRHKVCEIHGKTTKALVGNQMQRFCQQCSRFHPLSEFDEGKRSCRRRLAGHNRRRRK
OsSPL16	LOC_Os08g41940.1	CAVDGCKEDLSKCRDYHRRHKVCEAHSKTPLVVVSGREMRFCQQCSRFHLLQEFDEAKRSCRKRLDGHNRRRRK
OsSPL17	LOC_Os09g31438.1	CQVEGCGVDLSGVKPYYCRHKVCYMHAKEPIVVVAGLEQRFCQQCSRFHQLPEFDQEKKSCRRRLAGHNERRRK
OsSPL18	LOC_Os09g32944.1	CAVDGCKADLSKHRDYHRRHKVCEPHSKTPVVVVSGREMRFCQQCSRFHLLGEFDEAKRSCRKRLDGHNRRRRK
OsSPL19	LOC_Os11g30370.1	CSVDGCRSDLSRCRDYHRRHKVCEAHAKTPVVVVAGQEQRFCQQCSRFHNLAEFDDGKKSCRKRLDGHNRRRRK
CNR	Solyc02g077920	CQVDQCTADMADAKPYHRRHKVCEFHSKSPIVLISGLQKRFCQQCSRFHLLAEFDDAKRSCRRRLAGHNERRRK
SlySBP2	Solyc04g045560	CQVDSCNLDLSSAKQYHRKHRVCHIHSKCPKVIILGRHRRFCQQCSRFHSLSDFDENKRSCRTRLSDHNARRRK
SlySBP3	Solyc10g009080	CQVEECTADMVNAKTYHRRHKVCEFHAKAPEVLIDGLRQRFCQQCSRFHQLAEFDDAKKSCRRRLAGHNERRRK
SlySBP4	Solyc07g053810	CQAEKCNVDLSDAKQYHKRHKVCEYHAKSQVVVVAGLRQRFCQQCSRFHELTEFDESKRSCRRRLAGHNERRRK
SlySBP6a	Solyc03g114850	CQVQGCGKDLSPCKDYHKRHKVCEVHSKTAKVIVNGIEQRFCQQCSRFHLLAEFDDGKRSCRKRLAGHNERRRK
SlySBP6b	Solyc05g012040	CQVHGCNKDLSSSKDYHKRHKVCNEHSKTAIVIVNGIEQRFCQQCSRFHLLAEFDEGKRSCRKRLAGHNERRRK
SlySBP6c	Solyc12g038520	CQVQGCGKDLTSCKDYHKRHKVCEIHSKTAKVIVNGIQQRFCQQCSRFHLLAEFDDGKRSCRKRLAGHNERRRK

SlySBP7	Solyc01g080670	CQVPDCEADISELKGYHKRHRVCLRCANATSVVLDGHSKRYCQQCGKFHILSDFDEGKRSCRRKLERHNNRRRR
SlySBP8a	Solyc10g018780	CQAEGCNADLTHAKHYHRRHKVCEFHSKASTVIAAGITQRFCQQCSRFHLLSEFDNGKRSCRKRLADHNRRRRK
SlySBP8b	Solyc01g090730	CQAEGCNADLTHAKHYHRRHKVCEFHSKASTVIAAGLTQRFCQQCSRFHVLSEFDNGKRSCRKRLADHNRRRRK
SlySBP10	Solyc05g015510	CQVEGCNLDLSSAKEYYRKHRVCDSHSKSPKVIVAGVARRFCQQCSRFHSVSEFDDKKRSCRRRLSDHNARRRK
SlySBP12a	Solyc01g068100	CQVDDCGTDLSKAKDYHRRHKVCEMHSKASRALVGNVMQRFCQQCSRFHALQEFDEGKRSCRRRLAGHNKRRRK
SlySBP12b	Solyc05g053240	CQVQDCRADLSSAKDYHRRHKVCEVHSKAAKALVGNVMQRFCQQCSRFHVLEEFDEGKRSCRRRLAGHNKRRRK
SlySBP13	Solyc05g015840	CLVDGCNADLSECREYHRRHKVCEVHSKTAKVTIAGRDQRFCQQCSRFHSLVEFDDGKRSCRKRLDGHNRRRRK
SlySBP15	Solyc10g078700	CQVEGCQADLSDAKAYYSRHKVCGMHSKSPTVVVAGLEQRFCQQCSRFHQLTEFDQGKRSCRRRLACHNERRRK
VvSBP1	XM_002273498.1	CQVHGCNMDLSSSKDYHKRHKVCDVHSKTPKVIVNGIEQRFCQQCSRFHLLAEFDDGKRSCRKRLAGHNERRRK
VvSBP2	XM_002271276.1	CQVEGCNLDLSTAKDYHRKHRVCESHTKCPKVIVGGLERRFCQQCSRFHSLSEFDEKKRSCRRRLSDHNARRRK
VvSBP3	XM_002270190	CLVDGCKSDLSKCRDYHRRHKVCELHSKTAKVTIGGHEQRFCQQCSRFHSLGEFDEGKRSCRKRLDGHNRRRRK
VvSBP4	NW_002239818.1	CQADDCGVDLRAAKRYHRRHKVCERHAKAAFVFLGGIEQRFCQQCSRFHEISQFDDTKRSCRKRLAGHNQRRRK
VvSBP5	XM_002274898	CQVEDCRADLGNAKDYHRRHKVCDMHSKASKALVGNVMQRFCQQCSRFHLLQEFDEGKRSCRRRLAGHNRRRRK
VvSBP6	XM_002277003.1	CQVTGCEADISELKGYHRRHRVCLRCANASVVILDGQNKRYCQQCGKFHILSDFDEGKRSCRRKLERHNNRRRR
VvSBP7	XM_002273192.1	CQVEDCGADLSKAKDYHRRHKVCEMHSKAGCALVGNDMQRFCQQCSRFHVLQEFDEGKRSCRRRLAGHNKRRRK
VvSBP8	XM_002278476.1	CQVEGCKVDLSDAKAYYSRHKVCGMHSKSPTVIVAGLEQRFCQQCSRFHQLAEFDQGKRSCRRRLAGHNERRRK
VvSBP9	XM_002280016.1	CQVDNCTADMSEAKRYHKRHRVCEHHAKAPVILIAGIQQRFCQQCSRFHELSEFDDTKRSCRRRLAGHNERRRK
VvSBP10	XM_002267188.1	CQVEGCNLDLKSAKDYHRRHRICENHSKSPKVIVAGLERRFCQQCSRFHELTEFDDKKRSCRRRLNDHNARRRR

VvSBP11	XM_002275692.1	CQAERCTADLTDAKQYHRRHKVCEHHAKAQVVVVGGIRQRFCQQCSRFHELSEFDEAKRSCRRRLAGHNERRRK
VvSBP12	XM_002274324.1	CLVDGCTSDLRNCREYHRRHRVCERHSKTPVVIIGGQEKRFCQQCSRFHSLGEFDEVKRSCRKRLDGHNRRRRK
VvSBP13	XM_002274466.1	CQVEGCHVALVNAKDYHRRHKVCEMHSKAPKVVVLGLEQRFCQQCSRFHAVSEFDDSKRSCRRRLAGHNERRRK
VvSBP14	XM_002277147.1	CQAEGCNADLTHAKHYHRRHKVCEFHSKASTVFAAGLTQRFCQQCSRFHLLSEFDNGKRSCRKRLADHNRRRRK
VvSBP15	XM_002280124.1	CLVDGCNSDLSNCREYHRRHKVCELHSKTAQVTIGGHTQRFCQQCSRFHSLEEFDEGKRSCRKRLDGHNRRRRK
VvSBP16	XM_002265167.1	CQVHGCNKDLSSSKDYHKRHKVCEVHSKTAKVIVNGIEQRFCQQCSRFHLLAEFDDGKRSCRKRLAGHNERRRK
VvSBP17	XM_002273748.1	CQVDNCREDLSNAKDYHRRHKVCEMHSKSTKALVGKQMQRFCQQCSRFHPLSEFDEGKRSCRRRLAGHNRRRRK
VvSBP18	XM_002282562.1	CQAEKCGADLTDAKRYHRRHKVCEVHAKAAMVEVAGLRQRFCQQCSRFHELSEFDEAKRSCRRRLAGHNERRRK
ZjSBP01	CM003114.1	CQADNCNADLTDAKHYHRRHKVCEFHAKAPVVLVAGHQQRFCQQCSRFHGLLEFDENKRSCRRRLAGHNERRRK
ZjSBP02	CM003117.1	CQVDNCKEDLSNAKDYHRRHKVCELHSKSTKALVAKQMQRFCQQCSRFHPLTEFDEGKRSCRRRLAGHNRRRRK
ZjSBP03	CM003117.1	CQAERCGADLSDAKRYHRRHKVCEFHFKAPAVIVAGLPQRFCQQCSRFHELSEFDEAKRSCRRRLAGHNERRRR
ZjSBP04	CM003118.1	CLVDGCKADLSTCRDYHRRHKVCELHSKTPQVTIGGNTQRFCQQCSRFHSLEEFDEGKRSCRKRLDGHNRRRRK
ZjSBP05	CM003118.1	CQVYGCNKDLSSSKDYHKRHKVCELHSKTAKVIVNGIEQRFCQQCSRFHLLAEFDNGKRSCRKRLAGHNERRRK
ZjSBP06	CM003118.1	CQVQGCNLDLKLAKDYHRRHRICEIHSKSPKVIVAGMERRFCQQCSRFHELSEFDGKKRSCRRRLSDHNARRRR
ZjSBP07	CM003120.1	CQVPTCGADIRELKGYHRRHRVCLRCANASTVVLDGETKRYCQQCGKFHLLSDFDEGKRSCRRKLERHNNRRRR
ZjSBP08	CM003121.1	CQADECGLDLKLAKPYHKRHKVCERHAKAAVVLVNGLRQRFCQQCSRFHEISEFDGNKKSCRERLAGHNLRRRK
ZjSBP09	CM003122.1	CQVEDCGADLSNAKDYHRRHKVCEMHSKASRALVGNVMQRFCQQCSRFHVLQEFDEGKRSCRRRLAGHNKRRRK
ZjSBP10	CM003123.1	CQVHGCNMDLSSSKDYHKRHKVCDVHSKTAKVIVNGIEQRFCQQCSRFHLLAEFDDGKRSCRRRLAGHNERRRK

ZjSBP11	CM003123.1	CQVEGCNLDLSSAKDYHRKHRICESHSKSPKVIVGGLERRFCQQCSRFHPLSEFDEKKRSCRRRLSDHNARRRK
ZjSBP12	CM003123.1	CLVDGCISDLSKCRDYYRRHKVCELHSKTPKVTIGGHEQRFCQQCSRFHSLEEFDEGKRSCRKRLDGHNRRRRK
ZjSBP13	CM003125.1	CQVEGCKVDLSDAKAYYCRHKVCGMHSKTPKVIVAGLEQRFCQQCSRFHQLPEFDQGKRSCRRRLAGHNERRRK
ZjSBP14	CM003125.1	CQVEDCRADLSNAKDYHRRHKVCDMHSKASKALVGNVMQRFCQQCSRFHVLKEFDEGKRSCRRRLAGHNRRRRK
ZjSBP15	KN813464.1	CQAEKCTADLTDAKQYHRRHKVCEIHAKAQVVVVGGLRQRFCQQCSRFHELSEFDETKRSCRRRLAGHNERRRK
ZjSBP16	KN815747.1	CLVDDCKADLSSCREYHKRHRVCERHSKTPTVMVKGEEKRFCQQCSRFHALGEFDEVKRSCRKRLDGHNRRRRK

Supplementary Table 2 Primer sequences of ZjSBP in quantitative real-time PCR.

Gene	Forward primer (5'-3')	Reverse primer (5'-3')
ZjSBP01	GAAGAGGAGGAGGATGAAG	TGGAACCTGCTACATTGT
ZjSBP02	GATGTTGTTGATGCCTTGA	AACCTTGTGAACCTGGAA
ZjSBP03	GTGCTGATCTGAGTGATG	AGTCTGTGTAGTCTGGATT
ZjSBP04	ACCATAGACGCCATAAGG	GAGGAATATACAAGTGAAGTAGAA
ZjSBP05	GTGAGAACAGTAGTCAATATCC	TGCCGTAACAAGTATGGT
ZjSBP06	TAGGAGACAGCAGAGGAA	CATTATCGGTTGACAGAAGAG
ZjSBP07	GATTGAGCAGAGACTTGATG	ACTACTTGACTTCGTGTTATTC
ZjSBP08	AAGAAGAAGAAGAAGAAGAAGAAG	ACCATTGACCAAGACTACG
ZjSBP09	CTTAACTCCACTCCACATTG	CACTGCTGCTGATGATTC
ZjSBP10	ATATACTTCCAGGTGCCATT	AGGTTCCAGATACAGATGATT
ZjSBP11	AGAGATGTAAGTCTTCTTGTCA	AGAGCCTTGTTGGATTCA
ZjSBP12	ATAGCCATTCTCAGTTAGGT	CCAATCTCCTTAGTATTATCAGTT
ZjSBP13	TGAGAGTCCTCCATCTGA	TCTTGTGAACTGCTGCTA
ZjSBP14	ATAGTCGCAGAGCAAGAA	CACCACAGAACACCAATC
ZjSBP15	GAGAGGAATAGGCAGAGAAT	CCACAACCACAACTTGAG
ZjSBP16	AACCTCAATCTTGGCATCT	GCTTCTCTTCACCTCATCA
Actin	GAGGAAGCAACTGGCAACTAAGG	TACGAGCAAGCTGGATATCCTTC
